# Mitochondria Transplantation from Stem Cells for Mitigating Sarcopenia

**DOI:** 10.14336/AD.2023.0210

**Published:** 2023-10-01

**Authors:** Xiulin Tian, Mengxiong Pan, Mengting Zhou, Qiaomin Tang, Miao Chen, Wenwu Hong, Fangling Zhao, Kaiming Liu

**Affiliations:** ^1^Department of Nursing, Second Affiliated Hospital, School of Medicine, Zhejiang University, Hangzhou, China.; ^2^Department of Neurology, Second Affiliated Hospital, School of Medicine, Zhejiang University, Hangzhou, Zhejiang, China.; ^3^Department of Neurology, First People’s Hospital of Huzhou, Huzhou, Zhejiang, China.; ^4^Department of Neurology, Zhuji Affiliated Hospital of Shaoxing University, Zhuji, Zhejiang, China.; ^5^Department of Neurology, Tiantai People’s Hospital of Zhejiang Province, Tiantai, Taizhou, Zhejiang, China.

**Keywords:** mitochondria, sarcopenia, stem cells, mitochondria quality control

## Abstract

Sarcopenia is defined as the age-related loss of muscle mass and function that can lead to prolonged hospital stays and decreased independence. It is a significant health and financial burden for individuals, families, and society as a whole. The accumulation of damaged mitochondria in skeletal muscle contributes to the degeneration of muscles with age. Currently, the treatment of sarcopenia is limited to improving nutrition and physical activity. Studying effective methods to alleviate and treat sarcopenia to improve the quality of life and lifespan of older people is a growing area of interest in geriatric medicine. Therapies targeting mitochondria and restoring mitochondrial function are promising treatment strategies. This article provides an overview of stem cell transplantation for sarcopenia, including the mitochondrial delivery pathway and the protective role of stem cells. It also highlights recent advances in preclinical and clinical research on sarcopenia and presents a new treatment method involving stem cell-derived mitochondrial transplantation, outlining its advantages and challenges.

## 1. Introduction

Sarcopenia is characterized by the degenerative loss of muscle mass and force, which can lead to a decrease in quality of life, an increased risk of fractures, and disability [1-3]. Aging is the main cause of sarcopenia, which affects nearly one-third of older adults [1, 4]. The prevalence of sarcopenia is estimated to be 19.8%, with a 7.9% prevalence of severe sarcopenia, which can result in a significant increase in healthcare expenses [5, 6]. Research has shown that sarcopenia increases the risk of death, self-care deficiencies, and falls [7, 8]. which is a major concern as the global aging population continues to grow. The prevalence of sarcopenia is likely to increase as the population ages [9].

Sarcopenia is a complex condition that is influenced by a variety of factors, including hormonal changes, activation of the inflammatory pathway, a decrease in physical activity, chronic illness, fat infiltration, poor nutrition, and motor neuron dysfunction [10]. Recent studies have found that mitochondrial dysfunction is a crucial factor in the progress of age-related sarcopenia [11]. A better understanding of the underlying causes of sarcopenia can help in the search for new and more effective treatments [12]. Currently, the main treatment for sarcopenia is to optimize nutrition and physical activity [13]. Physical activity, particularly medium-intensity training, has been shown to regulate mitochondrial networks and improve the inflammatory response by activating signals in the skeletal muscle [14]. Exercise can also improve the function of mitochondria by increasing markers of mitochondrial fusion, fission, and biogenesis [14]. However, there are limitations to exercise-based treatment, such as limited benefits for inactive patients, the absence of standardized approaches, and poor patient compliance.

**Figure 1.**
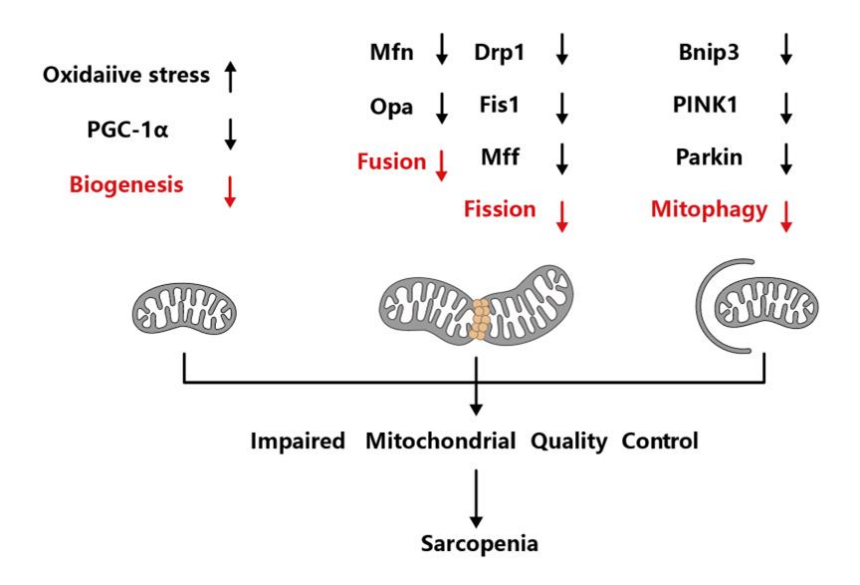
The potential pathogenetic mechanism of sarcopenia. Impaired mitochondrial biogenesis, dynamics and mitophagy have been regarded as the major molecular mechanisms in mitochondrial dysfunction, which could lead to the onset and progression of sarcopenia. Drp 1, dynamin-related protein 1; Mfn, mitofusin; OPA1, optic atrophy 1; PINK1, putative kinase 1; PGC-1α, peroxisome proliferative activated receptor-γ coactivator-1α; Fis1, fission protein 1; Bnip3, Bcl2 interacting protein3. (Figure created with BioRender.com).

Mitochondria are generally considered the cell's energy pool as they continuously produce ATP to maintain normal cell function, regulate cell proliferation, and Ca^2+^ stability, as well as integrate apoptotic signals [15]. Mitochondrial quality control (MQC) involves biogenesis, fusion, fission, and autophagy, and multiple cytokines and molecular signals work to preserve mitochondrial integrity. Mitochondrial biogenesis is a complex process that involves the interaction of mitochondrial and nuclear genes to produce new mitochondria. Mitochondrial dynamics degrade damaged mitochondria and generate new ones through continuous transformation between fusion and division, while autophagy selectively removes dysfunctional and injured mitochondria. If mitochondrial quality control fails, it may lead to mitochondrial dysfunction and muscle degradation (Fig. 1) [16]. The accumulation of injured mitochondria can result in the death of motor neurons and muscle fibers, indicating their importance in the development of sarcopenia [17]. This article reviews current research on mitochondrial transfer and its protective mechanisms and aims to provide an overview of the latest developments in stem cell therapy for age-related sarcopenia.

## 2. Mitochondria quality control in sarcopenia

MQC is a complex network in eukaryotes and encompasses four processes: mitochondrial proteostasis, biogenesis, dynamics, and mitophagy [18]. Maintaining healthy quality control is essential for preserving muscle cell homeostasis with aging and is crucial for maintaining cellular homeostasis [18]. Damaged mitochondrial biogenesis, dynamics, and autophagy are potential mechanisms of mitochondrial dysfunction that may contribute to the development of sarcopenia.

Many studies have shown that the delivery of mitochondria from stem cells to damaged cells is a new and promising therapeutic strategy for tissue injury and can be applied to a larger patient population [19, 20]. However, the specific mechanisms and key factors of this process are still being determined and the repeatability and effectiveness of the results from animal experiments need to be further tested in clinical trials [21]. Stem cells help degrade and clear damaged mitochondria, thereby improving cellular multiplication and anti-apoptotic function [19]. It has recently been discovered that stem cells transfer functional mitochondria to damaged cells, improving their aerobic respiration and restoring their metabolism [22]. This is considered a novel treatment for tissue injury [23, 24], particularly for mitochondrial disorders [25].

## 3. Types of stem cells

Stem cells can continuously replicate and differentiate into mature somatic cells [26]. Research on the transplantation of muscle-derived or non-muscle derived cells as a treatment strategy for skeletal muscle reconstruction has become a hot topic [27-31]. Myogenic cells, including satellite cells, can be quiescent or activated and are used to supplement the host tissue and produce stable progeny. Another type of cell, myoblasts, can be separated from autologous muscle tissue and used in vitro for culture. Muscle-derived stem cells can be divided into fibroblasts or tendon cells based on their cell capacities. Pericytes and intervascular cells can differentiate into muscle fibers and function through paracrine mechanisms. Among the non-myogenic cells, bone marrow mesenchymal stem cells are the most widely used in regenerative medicine and among the most promising. As a multipotent stem cell, MSCs can be acquired from various sources, such as bone marrow, fatty tissue, and the umbilical cord [32], and they promote stem cell growth, angiogenesis, motility, and differentiation through paracrine signals and/or immunomodulation. In recent years, induced pluripotent stem cell technology has made it possible to convert somatic cells into stem cells in vivo [33, 34].

## 4. Mechanisms and preclinical evidence of stem cell therapy in sarcopenia

Current strategies have mainly focused on nutritional regulation, cognitive training, and exercise to alleviate the development and symptoms of frailty. At present, there are no specific drugs that can be used to prevent and treat sarcopenia [35, 36]. Cell-based treatment, however, is a promising intervention to rescue the process of sarcopenia, and mesenchymal stem cells (MSCs) are the most potential candidates in regenerative therapy [37]. MSCs have been considered to ameliorate sarcopenia in many studies and have been transplanted to frail individuals. These cells act on the injured site, relieve inflammation, and restore cellular function [38]. It is worth noting that MSCs can improve the prognosis of frail patients by reducing tumor necrosis factor alpha (TNF-α) levels and inflammation and are a reliable option for individuals [38]. MSCs also play a role in the regeneration of injured tissues through mechanisms such as paracrine signals and direct differentiation.

**Table 1:** Clinical/preclinical study of stem cell mitigating muscular atrophy.

Cell source	Study type	Major findings	Ref.
ADSCs	Preclinical study, in vitro and in vivo	Improve motor function as well as decrease in senescence markers in old mice	39
myoblasts, myotubes and myofibres	Preclinical study, in vitro and in vivo	Enhance the rate of muscle regeneration and restoration of muscle function of mice	40
UC-MSCs	Preclinical study, in vitro and in vivo	Improve muscle atrophy and dysfunction in mice	41
hMSCs	Clinical trial	Improvements in motor function in patients with muscular atrophy	42
SMPs	Preclinical study, in vitro and vivo	Improving muscle histology and contractile function in mice	43
hMSCs	Clinical trial	Remarkable improvements in physical performance and motor function in patients with muscular atrophy	44
MSC	Clinical trial	Neurotrophic factors increased and inflammatory biomarkers decreased in patients with muscular atrophy	45
WJMSCs	Preclinical, in vitro	Improves mitochondrial functions and cellular performance in fibroblasts	46

Abbreviations: ADSCs adipose mesenchymal stem cells, UC-MSCs umbilical cord-derived mesenchymal stromal cells, hMSCs human mesenchymal stem cell, SMPs skeletal muscle precursors; IV intravenously, WJMSCs Wharton's jelly mesenchymal stem cells

There is some preclinical evidence that suggests stem cells can treat sarcopenia. Mitochondrial transplantation is deemed to a novel approach for the treatment of various forms of mitochondrial myopathy [19, 22]. Many studies have suggested that the transfer of mitochondria from stem cells to damaged cells can improve energy metabolism, preserve mitochondrial function, and improve quality of life (Table 1) [39-46]. Mitochondrial DNA (mtDNA) deficiency or DNA mutations cause a gradual decline in mitochondrial function and sarcopenia-related skeletal muscle atrophy. MSCs have the significant capability to deliver their own mitochondria to adjacent cells to cope with injury and apoptotic stress [47]. Mitochondrial transplantation from stem cells plays an active role in the in vitro study of mitochondrial encephalomyopathy [25, 46]. Evidence from cell culture, animal studies, and clinical data has shown the benefits of mitochondria transplantation in improving sarcopenia [48-50]. Mitochondria-rich stem cells that maintain structural and functional integrity can be obtained through amplification in vitro. Stem cell-based mitochondrial transplantation can be performed by directly transplanting stem cells into patients or by extracting mitochondria-rich extracellular vesicles (M-EVs) from stem cells and implanting them into patients. In vivo, the injection of M-EVs has been shown to enhance intracellular energy supply and restore muscle function [51]. In the case of genetic defects in fatal muscle diseases such as Duchenne muscular dystrophy, myogenic stem cell therapy using cell transplantation has been extensively investigated [31]. These positive studies provide a new perspective for sarcopenia intervention and suggest the potential role of MSC therapy. The substitution of dysfunctional mitochondria with functional exogenous mitochondria is considered a general principle for dealing with these diseases. As the basic understanding of cell behavior and its mode of action improves, cell therapy creates a new paradigm [52, 53].

The discovery that MSCs can donate their mitochondria to cells with abnormal mitochondrial function has opened up new direction for the treatment of sarcopenia related to mtDNA. Skeletal muscle mitochondrial dysfunction is considered the primary cause of sarcopenia [11], making stem cell transplantation a promising approach for treating the condition [27]. Stem cells are considered the best source of mitochondria for transplantation because they can enhance cell proliferation, provide resistance to oxidative stress, prevent apoptosis, and stimulate mitochondrial biogenesis [19]. As a result, stem cell-derived mitochondrial transplantation holds great promise for treating sarcopenia and could lead to the introduction of effective cell therapy into clinical practice.

The transfer of mitochondria between cells involves three steps. First, injured cells need to release signals that trigger mitochondrial transfer. Second, a mechanical structure needs to be formed to facilitate transmission. Third, the mitochondria are delivered to recipient cells, where they play a protective role [54].

## 5. Signals triggering mitochondrial transfer of stem cells

The signals that trigger the delivery of mitochondria from mesenchymal stem cells (MSCs) have long been a topic of interest among scholars. MSCs have the ability to detect changes in their environment, suggesting that the local microenvironment of damaged cells plays a key role in activating the signals for mitochondrial transfer [55, 56]. MtDNA from damaged cells is taken up by MSCs, triggering the cytoprotective effect of MSCs, improving mitochondrial biogenesis through retrograde signals, and preparing the MSCs to donate their mitochondria [57]. Furthermore, oxidative stress-regulated signaling mechanisms that participate in inflammation and tissue damage also trigger the transfer of mitochondria [49, 55, 58]. Previous research has suggested that the transfer of mitochondria from dysfunctional cells to MSCs relies on tunneling nanotubes (TNTs) [59]. Oxidative stress-induced apoptosis promotes the formation of TNTs [60], which may explain why cells in co-cultures that are injured transmit more mitochondria to MSCs. Thus, during the process of retrograde signal transduction, the levels of reactive oxygen species (ROS), calcium, and the AMP/ATP ratios in cells undergoing oxidative stress may trigger retrograde signal transduction and further promote the migrate of mitochondria from MSCs to the injured site [61].

Other studies have shown that astrocytic release can be induced by a ca-dependent mechanism involving CD38 and cyclic ADP ribose signaling, and that the transfer of astrocytic mitochondria to neurons contributes to neuroprotection and neurorestoration after stroke [62]. Additionally, mitochondrial proteins such as ROS, mtDNA, cardiolipin, or extracellular ATP are also involved in the inflammatory process and can induce the transfer of mitochondria from stem cells to recipient cells [63]. The above research indicates that multiple signaling molecules are involved in the delivery of mitochondria from MSCs, while the specific mechanism and signaling molecules that are released, as well as the downstream signals that are subsequently activated, have not been clearly defined.

## 6. Pathways of mitochondrial delivery

**Figure 2.**
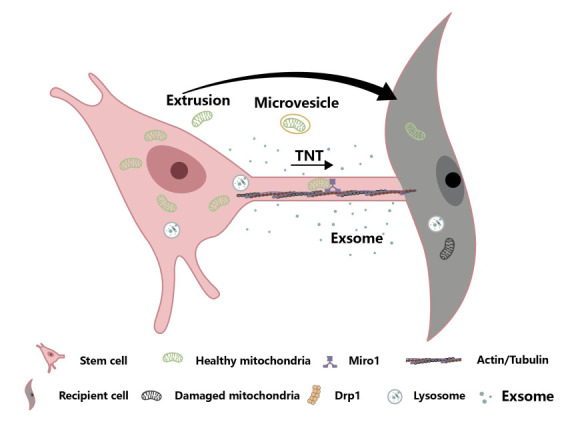
Different ways and protective mechanisms of mesenchymal stem cell mitochondrial transfer to damaged cells. Pathways by which healthy mitochondria are transferred from stem cells to mitochondrial dysfunction receptor cells include TNT formation, release of extracellular vesicles, and mitochondrial extrusion. Exosomes may transfer organelle fragments (such as protein complexes of mitochondrial electron transfer chains), mtDNA and ribosomes. Miro, mitochondrial Rho-GTPase1; TNT, tunneling nanotube; Drp 1, dynamin-related protein 1. (Figure created with BioRender.com).

The first stage of the mitochondrial delivery process involves the release of mitochondria from stem cells. There are several main mechanisms for intercellular mitochondrial delivery, including the formation of tunneling nanotubes (TNTs), the release of extracellular vesicles, cell fusion, and cell extrusion (Fig. 2). In order to increase the efficiency of stem cell transplantation, it is crucial to understand the mechanisms regulating these pathways [22].

### 6.1 Extracellular vesicles

The mtDNA of MSCs is transferred through extracellular vesicles (EVs), which alter the metabolism and inflammatory response of the recipient cells. Research has shown that mtDNA is transported through exosomes [64]. EVs derived from autologous stem cell cardiomyocytes have been shown to restore energy levels in the ischemic myocardium, potentially through the transfer of mitochondrial or key mitochondrial gene mRNA [51]. MSCs can deliver functional mitochondria into EVs, thereby increasing the mitochondrial content and function in skeletal soft tissues [65].

### 6.2 Cellular fusion

It has been reported that complete cell fusion can result in mitochondrial transfer. This process occurs by selectively losing the donor nucleus [66]. The stages involved in cell fusion include autophagy and the integrated stress response, rearrangement of the cytoskeleton, expression of fusogenic proteins, expression of proinflammatory cytokines, and intercellular contacts [67]. Stem cells can fuse with various cell types, including cardiomyocytes, respiratory epithelial cells, neurons, and hepatocytes [66, 68]. Previous research has shown that stem cells can rearrange myocardial cells to an immature phase through cell fusion and mitochondrial delivery [69]. However, both in vivo and in co-culture studies suggest that cell fusion may not be a major pathway for mitochondrial transfer and cell-based therapy [58, 69].

### 6.3 Tunneling nanotubes

Tunneling nanotubes (TNTs) are a type of transient tubular connections based on F-actin that allow for the direct exchange of various materials and signals between non-adjacent cells, including proteins, RNAs, organelles, and cytoplasm [70]. Membrane binding proteins have been found to be delivered intercellularly through TNTs [71]. Mesenchymal stem cells (MSCs) are often applied in co-culture systems to observe the viability of TNTs. TNTs can form between cells that are far apart, with a maximum distance of 150mm [71]. There are two types of TNTs: thin and thick [72]. TNTs are a novel means of interaction between cells for the transfer of mitochondria and macrophages, but not all cell types use TNTs for this purpose and the type of TNT and cargo transported can vary. Gap junctions are also involved in the transfer of mitochondria from bone marrow-derived mesenchymal stem cells to motor neurons [73].

Most scholars believe that complete mitochondrial transfer occurs through active transport and the formation of TNTs between donor and recipient cells [74, 75]. Factors that have been found to be related to mitochondrial delivery along TNTs include transport complexes, Miro1 and Miro2 Rho-GTPases, and TNFα/NF-κB/TNFαip2 signaling [49, 61, 76], Miro1 has been found to play a key role in the transfer of mitochondria via TNTs, as its expression can improve the metabolic benefits of MSC co-culture in cardiomyopathy [49], while reducing its expression can inhibit the formation of TNTs. Additionally, TNTs may be related to calcium signal transduction [77] and gap junctions can also facilitate the transfer of mitochondria between cells [78]. The gap junction channel, connexin-43, has been observed to facilitate the transfer of mitochondria from bone marrow-derived stromal cells to epithelial cells [55], but altering its shape can decrease the transfer of mitochondria.

## 7. Protection mechanism on recipient cells

**Figure 3.**
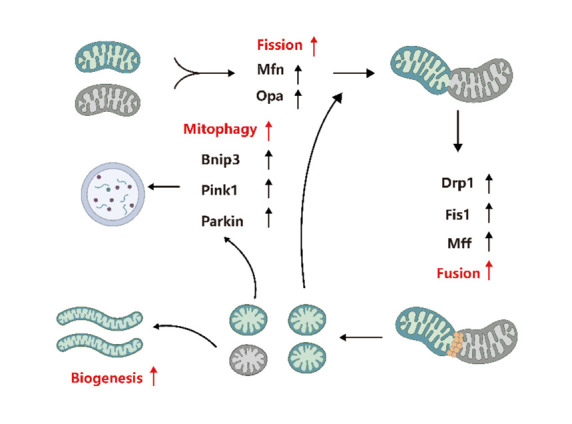
Stem cell-derived mitochondria may regulate mitophagy and mitochondrial biogenesis and optimize mitochondrial fission and fusion. (Figure created with BioRender.com).

After intercellular transmission, functional mitochondria can enter recipient cells and carry out specific functions while integrating with the cell's endogenous energy metabolism network [58]. Stem cell metabolism and differentiation play a role in mediating mitochondrial transfer. Evidence suggests that mitochondria from stem cells can increase the viability of recipient cells by regulating mitochondrial dynamics, acting as a biological engine that optimizes mitochondrial biogenesis and mitophagy, and promoting anti-inflammatory responses (Fig. 3).

### 7.1 Regulating mitochondrial biogenesis

Mitochondrial biogenesis is a multi-step process that creates new mitochondria [79]. This complex process involves the duplication of nuclear genes, transcription and translation of mtDNA, and protein import [80]. PGC-1α coactivates nuclear transcription cofactors (Nrf-1 and Nrf-2) and other downstream nuclear respiratory factors to regulate mitochondrial biogenesis [81]. The regulation of mitochondrial biogenesis and its factors play a role in the pathophysiologic variations caused by sarcopenia. A large study on human sarcopenia found that the expression profiles of PGC-1α, ERRα, and other coactivators in individuals with sarcopenia were reduced [82]. PGC-1α is crucial for maintaining muscle homeostasis and has received significant attention due to its potential role in age-related conditions [18]. In mammalian cells, PGC-1α acts as a link between the nucleus and mitochondria in mitochondrial biogenesis [83] by moving from the cytoplasm to the nucleus and mitochondria [83] Importing stem cell source mitochondria and their components can quickly augment mitochondrial biogenesis, and exogenous mitochondria must also integrate with the recipient cell's intracellular network. Data have shown that stem cell source mitochondria can enhance the viability of recipient cells by regulating mitochondrial biogenesis (Fig. 3).

### 7.2 Optimizing mitochondrial fusion

Mitochondrial dynamics involve the processes of mitochondrial fusion and fission. An imbalance in these dynamics can negatively impact mitochondrial health. This imbalance is a common feature of senescence [84] and has been observed in elderly individuals, where the levels of mRNA and the number of important fusion and fission proteins in skeletal muscle are lower compared to young individuals. Mitochondrial dynamics and autophagy play a key role in maintaining the health of mitochondria by preventing and repairing damage. There is evidence suggesting that stem cell-derived mitochondria may participate in the mitochondrial dynamics of sarcopenia receptor cells [54].

Mitochondrial fusion is facilitated by three large GTPases in the dynein superfamily: Mitofusin 1 (Mfn1), Mfn2, and Optic Atrophy 1 (Opa1). Mfn1 and Mfn2 are outer-membrane proteins that mediate outer-membrane fusion, while OPA1 has multiple intima-related isomers that mediate intima fusion [85]. The fusion process is important for oxidative phosphorylation (OXPHOS) activity, particularly in regulating mtDNA levels. The downregulation of Mfn2 and Opa1 expression in sarcopenia muscle suggests that mitochondrial fusion participate in the pathogenesis of sarcopenia [82]. Disrupting Mfn1/2 in skeletal muscle hinders mitochondrial fusion, leading to an accumulation of mtDNA defects and muscle atrophy [86]. These findings highlight the importance of abnormalities in mitochondrial fusion in muscle senility and sarcopenia, although the underlying mechanisms are not yet fully understood. Another study found that stem cells may protect against mitochondrial dysfunction in mice by transmitting mitochondria and increasing the expression of fusion genes OPA1, Mfn1, and Mfn2 in host cells [87].

### 7.3 Optimizing mitochondrial fission

Mitochondrial fission is regulated by the dynamin-related protein 1 (Drp1), a large GTPase that is recruited to the mitochondrial outer membrane through the action of collecting receptor proteins (Mff and Fis1). In addition to impacting mitochondrial shape, fission also plays a role in various functions, including promoting mitochondrial transport, mitophagy, and apoptosis [85]. Currently, studies have shown that the mechanism related to mitochondrial dynamics in aging muscle is weakened or missing. The knockout of fusion proteins (Mfn or OPA1) or fission proteins (Drp1, Fis1, and Fis2) in stem cells disrupts the normal functioning of the mitochondrial network and even alters the stem cell characteristics. Stem cells can regain a dysfunctional mitochondrial network and unusual fusion/fission proteins in MERRF hybrid cells by delivering mitochondria [25].

### 7.4 Regulating mitochondrial mitophagy

Mitochondrial mitophagy is a form of autophagy that helps to maintain the balance of mitochondria by selectively removing damaged or excess organelles and protein clusters [88, 89]. Insufficient autophagy leads to the persistence of dysfunctional mitochondria in skeletal muscles and neurons. However, autophagy decreases with aging in both skeletal muscles and motor neurons. The PINK1/Parkin pathway is currently considered a potential target in regulating ubiquitin-dependent mitophagy [90]. Studies have shown that the regulators of mitosis change with aging [91], leading to a decline in autophagy and an increase in abnormal mitochondria in skeletal muscles [92]. The accumulation of damaged mitochondria causes a decline in skeletal muscle function, resulting in muscle wasting and decreased muscle strength [93]. The loss of Parkin impairs muscle content and performance in the elderly and in mice [92, 94], while its overexpression can improve skeletal muscle health and alleviate sarcopenia [94, 95].

Mitochondria-derived vesicles play a key role in the transport of vesicles between mitochondria and lysosomes and are thought to be a pathway of Drp1-independent autophagy [96]. Additionally, nicotinamide riboside derived from these vesicles may serve as a new marker for sarcopenia [96].

In summary, autophagy and mitochondrial dynamics play a crucial role in maintaining a healthy mitochondrial network by facilitating the separation and subsequent degradation of decaying mitochondria [97]. PINK1 speeds up the degradation of flawed mitochondria by indirectly activating Drp1 [98]. Parkin induces the degradation of mitotic proteins by the proteasome, leading to mitochondrial fission, inhibiting mitochondrial fusion, and separating dysfunctional mitochondria from functional networks [99]. Given the many benefits of autophagy in quality control, it is promising to make Parkin or other key factors that regulate autophagy a target for preventing and reducing sarcopenia during aging. Existing data suggests that stem cell-derived mitochondria may improve the viability of recipient cells through mitochondrial mitophagy.

### 7.5 Acting as a “bioengine”

Mitochondria are commonly referred to as the powerhouses of cells. They produce and store chemical energy in the form of adenosine triphosphate (ATP). Additionally, mitochondria are a significant source of ROS, particularly through electron leakage from complexes I and III, and play a role in autophagy and cellular apoptosis [100]. With aging, there is an elevate in ROS accumulation, and an elevate in specific ROS levels has been shown to contribute to cellular senescence [101, 102]. However, mitochondrial transfer has been shown to reverse this trend. Bone marrow-derived mesenchymal stem cells (MSCs) have been suggested to improve energy metabolism in cells under oxidative stress by delivering functional mitochondria [103].

A study by Perico et al. [104] used a mouse model of acute kidney injury to show that healthy MSC transplantation could restore the viability of damaged tubular cells and replenish their energy production capacity through mitochondrial transfer. The transfer of mitochondria from stem cell sources may also contribute to adjusting mitochondrial biogenesis in host cells with mitochondrial dysfunction.

### 7.6 Promoting immune function and anti-inflammatory response

The immune-regulatory function of stem cells is achieved through paracrine mechanisms and cell-to-cell communication. Stem cells can regulate a varity of immune cells, such as T cells, B cells, natural killer cells, and macrophages, through cytokines they secrete [105]. Jackson MV et al. found that stem cells could transfer mitochondria to host macrophages, thereby boosting macrophage phagocytosis and bioenergetics, and improving pathogen clearance [106]. Through the delivery of mitochondria, stem cells also secrete exosomes containing microRNAs. After being ingested by macrophages, these microRNAs can inhibit the pro-inflammatory response by targeting the Toll-like receptor (TLR)/NF-κB pathway [107].

Another discovery of stem cells' immunomodulatory influence is that they can inhibit airway inflammation in asthma models by transferring mitochondria to stressed epithelial cells. This transfer is believed to be regulated by Miro and to rely on the interaction of Miro1 and Kinesin, which is calcium-sensitive. Miro1 overexpressing stem cells have been found to have better therapeutic effects in improving epithelium-mediated immune response amplification by enhancing mitochondrial donation [108]. The injection of IL-6 and TNF-α into rats has been found to cause skeletal muscle degradation due to the tissue damage or degeneration it causes in combination with inflammation [109]. Inflammatory factors lead to mitochondrial dysfunction, which reduces ATP production and increases reactive oxygen species (ROS) production [86]. Excessive ROS production can further worsen mitochondrial injury and metabolic disorders, leading to skeletal muscle atrophy through enhancement of the ubiquitin-proteasome system, a key route of protein degradation [110]. Zhang et al. [49] found that the pro-inflammatory cytokine TNF-α is involved in the regulation of the TNF-α/NF-κB/TNF-αip2 signaling pathways, leading to the polymerization of F-actin and the generation of TNTs by actin-driven plasma membrane protrusions of MSCs. The temporal regulation of cytokine levels is likely related to different stages of the immune response. The increased production of pro-inflammatory cytokines, such as TNF-α, can trigger the formation of TNTs and facilitate mitochondrial transfer in the early stages of the immune response. In the later stages of the immune response, stem cells may slow down mitochondrial metastasis by down-regulating cytokines through paracrine mechanisms. This highlights the importance of using stem cells at the correct time and under the correct conditions [54].

### 7.7 Improving cell viability

Mitochondria play an important role in regulating cell death [111]. The main product of mitochondrial metabolism, reactive oxygen species (ROS), has a important impact on mitochondria and mitochondria-regulated apoptosis [112]. Usually, the first step in apoptosis is the activation of Bcl-2 family proteins and the depolarization of mitochondria. Bcl-2 passes through the outer mitochondrial membrane, disrupting the electrochemical gradient in the inner mitochondrial membrane. This leads to the destruction of mitochondrial membrane features and a shortage of ATP production, as well as the activation of specific apoptotic proteases, such as caspases [54].

Aging can lead to the loss of succinate dehydrogenase and cytochrome c oxidase in mitochondria. This can result in mitochondrial rupture and an increase in apoptotic protein caspase-3 in the same muscle fibers. Caspase-3, an executor of apoptosis, triggers the early stages of apoptosis [113]. Bcl-2 can prevent the translocation of cytochrome c in mitochondria, thereby intercepting caspase activation and the apoptotic process. An imbalance in the Bax/Bcl-2 ratio is a common characteristic of apoptosis.

Stem cell mitochondrial transfer can promote cell viability [114] by adjusting the Bax/Bcl-2 balance and reducing the expression of caspase-3, which reduces the apoptosis level of recipient cells [103]. Interestingly, the delivery of dysfunctional mitochondria from injured cells to stem cells can also affect the stem cells.

## 8. Future prospects and challenges

Stem cells have several desirable characteristics such as low immunogenicity, long-term proliferation, and an increase in the number of mitochondria [19]. The transfer of mitochondria from stem cells to injured cells is a promising new target, regardless of the type of stem cell used. However, there are several challenges that need to be addressed before stem cell therapy can be successfully translated from experimental to clinical applications.

First, how to isolate complete and functional mitochondria from stem cells is a crucial challenge. Although intact mitochondria can help to restore cell function, the transfer of damaged or dysfunctional mitochondria can initiate an innate immune response when mtDNA is localized outside the mitochondrial matrix. Improving the techniques used to obtain high-quality active exogenous mitochondria will help to achieve the therapeutic goal [19].

Second, it is important to determine the source of mitochondrial injury that triggers apoptosis. ROS damage to the inner membrane and mtDNA of mitochondria may affect mitochondrial function and induce aging in muscle cells. However, mitochondrial injury can also be caused by post-translational modifications such as ubiquitination [115], acetylation [116], succinylation [117] and phosphorylation [118]. Determining which pathways can lead to reversible damage and which pathways result in irreversible injury and apoptosis is crucial.

Third, there are several pathways of mitochondrial transfer including TNTs, EVs, and gap junction channels. Each of these pathways has different signaling pathways and it is not yet clear if cells can transfer mitochondria through multiple pathways simultaneously [119]. Additionally, the selection of the method of transfer needs to be defined and standardized.

Fourth, maintaining the activity of enough quantity of mitochondria in vitro is also a challenge. It is not yet known if the cryopreservation of mitochondria is as effective as freshly isolated mitochondria.

Fifth, current strategies to reduce sarcopenia through physical activity and nutrition need to focus on improving mitochondrial health. Future research should examine how exercise and nutrition can improve mitochondrial health and reverse the mitochondrial changes that occur during aging. The accumulation of ROS may increase with exercise and may partly explain why exercise can only partially reduce muscle loss. The addition of antioxidants to reduce excessive ROS accumulation, oxidative injury, and opening of the mitochondrial inner membrane pore during exercise may also be explored in future research.

Therapeutic studies in the future should focus on improving the drug strategy for stem cell-derived mitochondrial transfer [58]. Understanding the molecular mechanisms, metabolic capability, dynamics, and quality control of transplanted mitochondria will be crucial to facilitate the application of stem cell therapy for mitochondrial encephalomyopathies [22]. Currently, there are limited animal experiments for sarcopenia based on mtDNA and most research on mitochondrial diseases is still in vitro. The development of fluorescence microscopy coupled with mitochondrial tracking tools, such as the recently developed MitoCeption [120], will help to further study the process of mitochondrial delivery in vivo. As the comprehension of the mechanisms of mitochondrial delivery increases and more preclinical studies are conducted, the gap between basic research and clinical applications will become increasingly narrow in the future.

## 9. Conclusion

Sarcopenia is a progressive aging-related disease that is associated with many negative consequences. It is important to note that age-related muscle loss can be prevented and treated, but a full understanding of the causes and mechanisms of sarcopenia is still lacking. Mitochondrial dysfunction in muscle cells is believed to be a major contributor to sarcopenia. Stem cell therapy, with its ability to renew itself and produce anti-inflammatory cytokines, may offer a new approach to treating sarcopenia. These cytokines can change the microenvironment and support nerve regeneration. Current research focuses on using stem cells as a source of mitochondria for transplantation, as this can provide functional, normal mitochondria to produce ATP, reduce inflammation, prevent apoptosis, and ultimately help to rescue damaged cells in conditions such as stroke, Parkinson's disease, neurological trauma, lung injury, and organ dysfunction following trauma [19, 22, 50, 54, 55, 121-123].

However, stem cell-based therapy can also have risks, so clinical applications of stem cells need to be evaluated carefully. Once technical problems are resolved, this new method of stem cell-based mitochondrial transplantation for treating sarcopenia is expected to be widely used.
